# Antitumor Necrosis Factor-Alpha (TNF-*α*) Infliximab-Induced Pleural Effusion and Pericarditis in Crohn's Disease

**DOI:** 10.1155/2021/9989729

**Published:** 2021-07-12

**Authors:** Ashley Fonseca, Julee Sunny, Lina M. Felipez

**Affiliations:** ^1^Department of Medical Education, Nicklaus Children's Hospital, Miami, FL, USA; ^2^Department of Pediatric Gastroenterology, Hepatology and Nutrition, Nicklaus Children's Hospital, Miami, FL, USA

## Abstract

Crohn's disease (CD) is a chronic inflammatory disease that can be associated with intestinal and extraintestinal manifestations. Some patients are treated with infliximab, an antitumor necrosis factor-alpha (TNF-*α*) agent, to help them achieve and maintain clinical and biochemical remission. However, some patients with CD can present severe adverse effects such as drug-induced lupus and rarely present with pleural space and pericardium involvement. We report a case of an 18-year-old Hispanic male with CD who acquired anti-TNF-*α*-induced lupus after infliximab therapy presenting with pleural effusion and pericarditis. The patient presented with a 2-week history of pleuritic chest pain. Initial laboratory workup was remarkable for leukocytosis and increased inflammatory markers. Imaging and cardiovascular studies were consistent with pericarditis and pleural effusions. Serositis was initially thought to be reactive secondary to the current *Mycoplasma pneumoniae* infection. He was treated with colchicine 0.6 mg PO TID for six weeks and azithromycin 500 mg PO for seven days. Pain improved after discharge but resurfaced on the day of infliximab infusion. Imaging and cardiovascular studies demonstrated the persistence of pleural effusions and pericarditis. Ultrasound-guided thoracentesis was consistent with exudative pleural effusions. Rheumatological workup was remarkable for increased antihistone antibodies, consistent with drug-induced lupus. Infliximab-induced pericarditis and pleural effusions are rarely reported in the literature. It is thought that infliximab may have a proinflammatory activity or have a delayed type III hypersensitivity reaction. The first line of therapy of anti-TNF-*α*-induced lupus is the withdrawal of the offending drug. Our patient is unique as few cases of anti-TNF-*α*-induced pleural effusion and pericarditis in CD are reported. After discontinuing the offending drug, ustekinumab was started, and maintaining a steroid and colchicine regimen, the patient's chest pain improved. Antihistone antibodies have returned to normal one month after starting ustekinumab.

## 1. Introduction

Crohn's disease (CD) is a chronic inflammatory disease associated with intestinal and extraintestinal manifestations [1]. It is characterized by chronic, relapsing transmural inflammation that can present as skip lesions throughout the gastrointestinal tract and manifests as chronic abdominal pain, diarrhea, obstruction, and/or perianal lesions. Its actual cause is unknown, but it is believed immunological, genetic, gut dysbiosis, and environmental factors are involved in the disease's onset and progression [2]. Biologic therapy such as infliximab, an antitumor necrosis factor-alpha (TNF-*α*) agent, has been shown to achieve and maintain remission and prevent a recurrence in 56% of the pediatric population [3, 4]. However, 6% of patients with CD can present with serious adverse effects such as drug-induced lupus following treatment with infliximab and rarely present with pleural space and pericardium involvement [5]. Here, we report a case of an 18-year-old Hispanic male with a stenotic, ileocolonic CD who acquired anti-TNF-*α*-induced lupus after infliximab therapy presenting with pleural effusion and pericarditis.

## 2. Case Report

**Figure 1:**
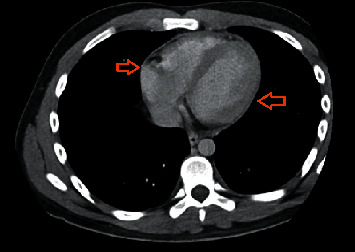
Computed tomography (CT) of the chest demonstrating pericardial thickening.

**Figure 2:**
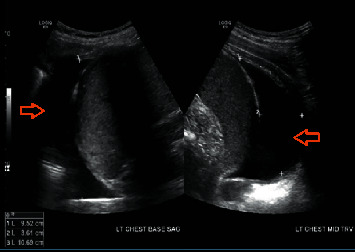
Ultrasound of the chest demonstrating a left simple pleural effusion measuring 150 mL.

An 18-year-old Hispanic male with a stenotic, ileocolonic Crohn's disease presented with a 2-week history of pleuritic chest pain. The pain was located at the mediastinum, radiating to the shoulder, worse with deep breaths and lying flat, and improved by standing up or sitting. No prior history of any autoimmune or atopic disease was noted in the patient and his family. CD management consisted of infliximab 10 mg/kg every four weeks, which was increased from 5 mg/kg after obtaining a serum infliximab (IFX) concentration of 2.1 *μ*g/mL and antibodies to infliximab (ATI) concentration of <3.1 U/mL on week 12. Prior medication history only included mesalamine and methotrexate. Laboratory workup on admission was remarkable for a leukocytosis of 13.2 10 k/uL and increased CRP of 8.2 mg/L, rheumatoid factor of 12.5 IU/mL, and calprotectin of 480 mcg/gm (Table 1). The electrocardiogram (EKG) showed nonspecific ST abnormalities. Computed tomography (CT) of the chest showed mild pericardial thickening with pericardial effusion and minimal bibasilar pleural thickening with bibasilar pleural effusions (Figure 1). An echocardiogram showed a small circumferential pericardial effusion. *Mycoplasma pneumoniae* IgM was positive, and he was initially treated with azithromycin IV 500 mg daily followed by 500 mg PO daily for a 7-day course treatment. Serositis was initially thought to be reactive secondary to the current *Mycoplasma pneumoniae* infection. Cardiology recommended colchicine 0.6 mg PO TID for six weeks. Partial rheumatological workup results were remarkable for negative antinuclear antibody (ANA) titers and positive antinuclear antibodies indirect fluorescent antibody (ANA IFA) (Table 1). Pain improved after discharge but resurfaced on the day of infliximab infusion. Antihistone antibodies previously obtained during his first admission resulted during his second presentation with increased levels of 1.7 U (Table 1). Chest X-ray showed blunting of the posterolateral left lateral costophrenic angle, suggestive of a small pleural effusion. EKG showed diffuse abnormal T waves. An echocardiogram showed an interval decrease in pericardial effusion. Ultrasound of the chest showed right and left simple pleural effusions, measuring 34 mL and 150 mL, respectively (Figure 2). Ultrasound-guided thoracentesis removed 430cc of clear amber fluid. Body fluid cultures were negative, and body fluid chemistry was consistent with an exudative pleural effusion. He was continued on colchicine 0.6 mg BID and discharged with a weaning schedule of prednisone 20 mg PO daily. Two weeks after the wean of steroids was started, his chest pain recurred and colchicine was increased to 0.6 mg PO TID and steroids were increased. Four weeks after discharge, he was started on an induction of ustekinumab 390 mg IV with a maintenance regimen of 90 mg SC. Serum ustekinumab (UST) concentration was 13.9 *μ*g/mL and antibodies to ustekinumab (ATU) concentration was <1.6 U/mL at week 12. Afterwards, the dose frequency was increased to every four weeks. Currently, the patient denies any chest pain or shortness of breath after discontinuing infliximab, starting ustekinumab, and adjusting his steroid and colchicine regimen during a two-month period after discharge. Antihistone antibodies returned to normal one month after starting ustekinumab.

## 3. Discussion

Anti-TNF-*α* therapy is effective in patients with inflammatory bowel disease (IBD) and other chronic inflammatory diseases. However, this therapy may be associated with the production of antinuclear antibodies (ANAs), double-stranded (ds) DNA antibodies, antihistone antibodies, and others, developing drug-induced lupus [6, 7]. Drug-induced lupus is a syndrome similar to idiopathic systemic lupus erythematosus (SLE).

The pathogenesis of anti-TNF-*α* in SLE is not entirely understood, but several hypotheses exist. One of them is called the “cytokine shift,” in which anti-TNF-*α* suppresses the production of Th1 cytokines, producing Th2 cytokines, IL-10, and INF-*α* instead and leading towards the production of autoantibodies and a lupus-like presentation [8, 9]. Another hypothesis predicts that inhibition of TNF-*α* can produce autoantibodies against DNA and other nuclear antigens by decreasing CD44 expression, thus affecting cellular apoptosis [8, 9].

Only a few cases of anti-TNF-*α*-induced lupus in IBD patients have been reported. Most patients present with rash, arthralgia, and fever. Rare manifestations are myalgias, pericardial/pleural effusion, glomerulonephritis, valvulitis, pneumonitis, deep vein thrombosis, and oral ulcers [7].

Infliximab-induced pericarditis and pleural effusions are rarely reported in the literature [1]. Drug-induced lupus with infliximab therapy in CD patients has only been seen in 0.6% to 1.6% of cases [10, 11]. Certain studies have also seen a presentation with antihistone antibodies in up to 57% of the patients with anti-TNF-*α* lupus [3]. It is thought that infliximab may have a proinflammatory activity or have a delayed type III hypersensitivity reaction [12].

The first line of therapy of anti-TNF-*α*-induced lupus is the withdrawal of the offending drug. Other patients may benefit from corticosteroids and immunosuppressive agents. After the offending drug is discontinued, autoantibodies have been seen to return to normal and symptoms have resolved [3].

Our patient is unique as few cases of anti-TNF-*α* infliximab-induced pleural effusion and pericarditis in patients with Crohn's disease are reported. After discontinuing the offending drug, ustekinumab was started, and maintaining a low-dose steroid and colchicine regimen, the patient's chest pain improved. Antihistone antibodies have returned to normal one month after starting ustekinumab.

## Figures and Tables

**Table 1:** Laboratory results of our patient upon presentation and diagnosis.

Parameter	Result	Normal range for age
White blood cells	13.2	5–10 k/uL
C-reactive protein (CRP)	8.2	0.0–1.0 mg/L
Rheumatoid factor	12.5	0–11.9 IU/mL
Calprotectin	480	<50 mcg/gm
Antinuclear antibodies (ANAs)	Negative	Negative
Antinuclear antibodies indirect fluorescent antibody (ANA IFA)	Positive	Negative
Antihistone antibodies	1.7	0.0–0.9

## Data Availability

The necessary data supporting this study are included within this paper.
